# Sociodemographics and their impacts on risk factor awareness and beliefs about cancer and screening: results from a cross-sectional study in Newfoundland and Labrador

**DOI:** 10.1186/s12889-020-09616-2

**Published:** 2020-10-06

**Authors:** Fuyan Shi, Lance Garrett Shaver, Yujia Kong, Yanqing Yi, Kris Aubrey-Bassler, Shabnam Asghari, Holly Etchegary, Kazeem Adefemi, Peizhong Peter Wang

**Affiliations:** 1 School of Public Health & Management, Weifang Medical University, Weifang, Shandong China; 2 Division of Community Health and Humanities, Faculty of Medicine, Memorial University of Newfoundland, St. John’s, Newfoundland and Labrador A1B 3V6 Canada; 3 Faculty of Medicine, University of British Columbia, Vancouver, BC Canada; 4 Dalla Lana School of Public Health, University of Toronto, Toronto, Ontario Canada

**Keywords:** Newfoundland and Labrador, Cancer, Awareness and beliefs, Risk factors, Health behaviours

## Abstract

**Background:**

Our objective was to examine cancer risk factor awareness and beliefs about cancer treatment, outcomes, and screening, and how these are mediated by sociodemographic variables, among Newfoundland and Labrador residents.

**Methods:**

Participants aged 35 to 74 were recruited through Facebook advertising, and a self-administered online questionnaire was used to collect data. Descriptive statistics, Spearman rank correlations, and multivariate logistic regression analyses were performed.

**Results:**

Of the 1048 participants who responded and met the inclusion criteria for this study, 1019 were selected for this analysis. Risk factor recognition was generally good, though several risk factors had poor awareness: being over 70 years old (53.4% respondents aware), having a low-fiber diet (65.0%), and drinking more than 1 unit of alcohol per day (62.8%). Our results showed that the participants’ awareness of risk factors was significantly associated with higher income level (*r*_*s*_ = 0.237, *P* <  0.001), higher education (*r*_*s*_ = 0.231, P <  0.001), living in rural regions (*r*_*s*_ = 0.163, P <  0.001), and having a regular healthcare provider (*r*_*s*_ = 0.081, *P* = 0.010). Logistic regression showed that among NL residents in our sample, those with higher income, post-secondary education, those in very good or excellent health, and those with a history of cancer all had higher odds of having more positive beliefs about cancer treatment and outcomes. Those with a history of cancer, and those with very good or excellent health, also had higher odds of having more positive beliefs about cancer screening. Finally, compared to Caucasian/white participants, those who were non-Caucasian/white had lower odds of having more positive beliefs about cancer screening.

**Conclusion:**

Among adults in NL, there was poor awareness that low-fiber diets, alcohol, and age are risk factors for cancer. Lower income and education, rural residence, and not having a health care provider were associated with lower risk factor awareness. We also found a few associations between sociodemographic factors and beliefs about cancer treatment and outcomes or screening. We stress that while addressing awareness is necessary, so too is improving social circumstances of disadvantaged groups who lack the resources necessary to adopt healthy behaviours.

## Background

Cancer is one of the most common causes of death and its incidence is increasing worldwide [1]. Recent findings from the Canadian Cancer Society show that for the four most commonly diagnosed cancers in Newfoundland and Labrador (NL)—prostate, lung, breast, and colorectal—the age-standardized incidence rates (ASIR) are considerably higher in NL compared to the rest of Canada [2]. There are large differences in mortality rates, as well. In 2019, the age-standardized mortality rates (ASMR) of all cancers in males and females in Canada were 222.8 and 166.0 per 100,000, respectively [2]. In NL, the ASMRs were 267.6 and 197.2 per 100,000 for males and females, respectively [2].

There are many factors, with complex mechanisms and interactions, affecting the incidence and mortality of cancer. Extensive research confirms that adopting healthy behaviours is both effective and economical in decreasing the risk of cancer [3]. Recent years have seen a strong focus on risk factor awareness and its possible role in cancer prevention [4–6]. Beliefs about cancer treatment and outcomes, as well as knowledge and beliefs about cancer screening, have been identified as important factors in cancer prevention, with regard to participating in screening and adopting healthy behaviours [7–10].

Studies in other countries have also shown that risk factor awareness and beliefs about cancer vary with socio-demographic variables [11, 12]. We also know from previous research in NL that inadequate intakes of a number of essential nutrients is common among older adults, and that dietary patterns vary by sociodemographic characteristics [13, 14]. There are a number of modifiable cancer risk factors which are common in NL, such as high risky alcohol use, low dietary fiber intake, low fruit and vegetable consumption, and high pickled red meat consumption [15–20].

It is therefore likely that risk factor awareness and beliefs about cancer play a role in NL’s disproportionately higher rates of cancer. Yet, it is not known how aware residents of NL are about the cancer risk associated with these modifiable factors, nor is it known what residents of NL believe about cancer and cancer screening. Therefore, it is important to assess risk factor awareness and beliefs about cancer and cancer screening among the general population in NL and to investigate their possible mediators, such as socioeconomic status. By understanding the sociodemographic factors which mediate risk factor awareness and cancer beliefs, this knowledge may serve as a basis for future health care practice and planning of targeted public health and social policy interventions.

To the best of our knowledge, no previous studies have been conducted to explore the level of cancer awareness and beliefs among Newfoundland and Labrador (NL) residents. We hypothesized that NL residents with different sociodemographic characteristics possessed heterogeneous levels of risk factor awareness, beliefs about cancer treatment and outcomes, and beliefs about cancer screening. The aim of this study was, therefore, to examine the level of risk factor awareness among NL residents ages 35 to 74, and to comprehensively investigate the independent sociodemographic factors associated with different levels of beliefs about cancer and cancer screening.

## Methods

### Study design and participants

This cross-sectional study was conducted among 1019 Newfoundland and Labrador residents aged 35 to 74 years old during the period from April to May 2018. All participants were recruited through Facebook advertising. The sample population was invited to complete a Web-based survey using a self-administered questionnaire. The process for recruiting the survey subjects is described in more detail in our previous publication [21].

### Study instrument

In our study, all data were collected through a purpose-built questionnaire using questions adapted from validated and reliable instruments, including the Awareness and Beliefs about Cancer (ABC) instrument [22] and Cancer Research UK’s Cancer Awareness Measure (CAM) [23]. The final questionnaire (see: Supplementary File 1) included sections on the following: (1) general physical and social well-being, (2) sociodemographic characteristics, including age, gender, ethnicity, educational level, annual household income, family history of cancer, having a regular healthcare provider, and number of chronic diseases; (3) awareness of cancer risk factors (11 items, 10 from the ABC instrument and 1 added by our team), (4) beliefs about cancer treatment and outcomes (6 items from the ABC instrument); and (5) beliefs about cancer screening (8 items, 3 adapted from the ABC instrument and 5 written by our team). The ABC instrument had one risk factor awareness question on physical activity that was omitted from our final questionnaire due to an error when transcribing the survey into the web survey tool.

To measure the overall awareness of risk factors for cancer, each correct answer scored one point, with the total possible scores ranging from 0 to 11 points. To assess the level of cancer beliefs about treatment and outcomes, participants were asked six questions (three in the positively-framed domain and three in the negatively-framed domain). Beliefs were scored on a four-point Likert-type scale (positively-framed domain: 1 = strongly disagree, 2 = disagree, 3 = agree, and 4 = strongly agree; and, for the negatively-framed domain: 4 = strongly disagree, 3 = disagree, 2 = agree, and 1 = strongly agree). To measure attitudes towards cancer screening, participants were asked eight questions (four questions in the positively-framed domain and four in the negatively-framed domain). These beliefs scored on a five-point Likert-type scale (positively-framed domain: 1 = strongly disagree, 2 = disagree, 3 = neither agree nor disagree, 4 = agree, 5 = strongly agree; and, for the negatively-framed domain: 1 = strongly agree, 2 = agree, 3 = neither agree nor disagree, 4 = disagree, and 5 = strongly disagree). Scores of individual items for beliefs scores were added together to create two composite variables (1) beliefs about cancer treatment and outcomes, and (2) beliefs about cancer screening. Higher composite scores indicated more positive beliefs.

### Statistical analyses

In our study, descriptive statistics for continuous variables were expressed as the mean ± standard deviation to illustrate participant characteristics. Frequencies and percentages were used to describe categorical variables. Age had 8 response categories, and income had 7 response categories that were combined into 3 categories (less than $30,000 ‘Low’ = 1; $30,000–79,999 ‘Middle’ = 2; and $80,000+ ‘High’ = 3) in addition to a “don’t know” response category (excluded from analysis). Individuals listed chronic disease they were diagnosed with, and these were counted and grouped into 3 categories (0; 1–2; and 3 or more chronic diseases). Awareness of individual risk factor items were scored as 0 (incorrect recognition) or 1 (correct recognition). Overall risk factor awareness was scored from 0 to 11 based on total number of individual items correctly recognized. Spearman rank correlation coefficients (*r*_*s*_) were used to measure the strength of the linear association between two ordinal characteristics (e.g., income level and risk factor awareness). We interpreted *r*_*s*_ effect size as 0.10 = weak, 0.30 = moderate, 0.50 = strong [24]. Multivariate logistic regression was used to look for associations between sociodemographic characteristics and beliefs about cancer treatment and outcomes as well as beliefs about cancer screening. Analysis with a two-tailed *P* value of less than 0.05 was considered statistically significant. All analyses were performed in SPSS version 21.0.

Our initial sample size calculation was based on primary outcome of cancer awareness (adequate/inadequate) assuming α = 0.05 and β = 0.2, respectively, and the required sample size was about 300. As we changed from a postal survey to an online survey due to budget constraint, we were able to collect data from 1019 eligible individuals.

## Results

### The social demographic characteristics of the participants

The study was approved by the Health Research Ethics Authority of Newfoundland and Labrador. Out of the 1019 participants, 23.3% (237/1019) were male, 95.4% (972/1019) were Caucasian/white, 77.5% (789/1019) had high educational level (any post-secondary, college or above), and 40.9% (378/1019) had high income (≥$80,000 per year). In total, 15.8% (161/1019) participants had a history of cancer, 69.0% (703/1019) had a family history of cancer in a first-degree relative, and 13.8% (141/1019) people had three or more chronic diseases. When asked to rate their current health, only 15.2% (155/1017) of people rated their health as poor or fair, 35.3% (360/1017) as good, and 49.9% (504/1017) reported that they were in very good or excellent health (Table 1). Among the participants, 29.5% (300/1018) of participants said they would make an appointment within a day to see a doctor or health care professional if they noticed symptoms they thought were serious, and 48.7% (496/1018) participants would seek a doctor within a week. Only 5.4% (55/1018) respondents indicated that they would wait more than a month to make an appointment, while 0.8% (8/1018) said they would not make an appointment.

**Table 1:** Social-demographic characteristics of the sample (*n* = 1019)

Variable	Number	Percentage (%)
**Age group (years)**		
35–39	96	9.4
40–44	97	9.5
45–49	125	12.3
50–54	158	15.5
55–59	167	16.4
60–64	166	16.3
65–69	140	13.7
70–74	70	6.9
**Gender**		
Female	779	76.4
Male	237	23.3
Other	3	0.3
**Ethnicity**		
Caucasian/white	972	95.4
Other	47	4.6
**Geography**		
Rural	452	44.4
Urban	567	55.6
**Annual household income (before taxes)**		
Low Income (<$29,999)	136	14.7
Middle Income ($30,000 - $79,999)	411	44.4
High Income ($80,000+)	378	40.9
Don’t know or missing	94	9.2
**Highest qualification obtained**		
Did not complete high school	38	3.7
High school	192	18.8
College diploma or university degree	602	59.1
Graduate, post-graduate, or professional degree	187	18.4
**History of Cancer (Self)**		
Yes	161	15.8
No	856	84.0
**History of Cancer (Significant Other**^a^**)**		
Yes	126	12.4
No	805	79.0
Not sure or missing	88	8.6
**History of Cancer (Close Family**^a^**)**		
Yes	703	69.0
No	284	27.9
Not sure or missing	32	3.1
**Self-rated health**		
Poor	15	1.5
Fair	140	13.7
Good	360	35.3
Very good	413	40.5
Excellent	91	8.9
**Number of chronic diseases**		
0	454	44.6
1–2	424	41.6
3 or more	141	13.8

^a^ Close family refers to parents, siblings, children, etc.; Significant other refers to husband/wife, partner, etc.

Our sample was partially representative of the population, and specifically, of rural and urban geography, age, income, proportion of people who have a regular healthcare provider, and of people who have had a colonoscopy or sigmoidoscopy [21]. However, our sample was over representative of women and those with a higher level of education; for a full analysis of representativeness and comparison with the underlying population, see Shaver et al. [21].

### Awareness of cancer risk factors

In our study, the risk factors which the greatest number of participants were aware of included smoking (97.44% [993/1018]), second-hand smoking (96.55% [980/1015]) and having a family history of cancer (90.53% [918/1014]). Interestingly, while 53.41% (540/1011) agreed that being “over 70 years of age” increased risk, when this question was asked in a different format earlier in the survey, only 12.58% correctly said that the “70 years-old” were the age group most likely to get cancer, while 2.46% (20/1017) believed it was 30 years-old, 30.97% (315/1017) of participants believed it was 50 years-old, and 53.98% (549/1017) thought people of any age were equally likely to be diagnosed with cancer. In addition, there was a low awareness of the risks associated with drinking more than 1 unit of alcohol per day (62.81% [635/1011]) and with having a diet low in fiber (65.02% [658/1012]) (Table 2).

**Table 2:** The correct recognition of risk factors by household income group

Risk factor	All (n, %)^c^	High income^**b**^ (*n* = 378)	Middle income^**b**^ (*n* = 411)	Low income^**b**^ (*n* = 136)	***r***_**s**_^**d**^	***P***-value
Smoking	993(97.54)	375(99.21)	400(97.32)	126(93.33)	**0.097**	**0.002**
Exposure to others’ cigarette smoke	980(96.55)	369(98.40)	395(96.34)	125(91.91)	**0.090**	**0.004**
Drinking more than 1 unit^a^ of alcohol per day	635(62.81)	267(71.01)	243(59.71)	68(50.37)	**0.118**	**< 0.001**
Eating less than 5 servings^a^ of fruit and vegetables	856(84.50)	329(87.04)	342(84.03)	111(82.84)	0.009	0.764
Eating red or processed meat once per day or more	705(69.25)	296(78.31)	261(63.50)	92(67.65)	**0.068**	**0.030**
Being overweight	826(81.54)	315(83.33)	339(83.50)	105(77.78)	−0.019	0.550
Getting sunburnt more than once as a child	860(84.48)	332(87.83)	346(84.18)	108(80.00)	0.032	0.311
Being over 70 years old	540(53.41)	245(65.16)	203(50.00)	54(39.71)	**0.109**	**0.001**
Cancer family history	918(90.53)	354(93.90)	364(89.00)	119(88.15)	0.047	0.132
Infection with HPV (human papillomavirus)	859(85.56)	328(87.23)	341(85.04)	112(82.96)	0.031	0.332
Having a diet low in fiber	658(65.02)	288(76.80)	255(62.50)	71(52.21)	**0.089**	**0.005**
Total score **(Median(Q1,Q3))**	9(7,10)	10(8,11)	9(7,10)	8(7,10)	**0.237**	**< 0.001**

^a^ 1 unit of alcohol = 1 can of beer/cider, or a 5 oz. glass of wine, or 1 oz. of spirits/hard alcohol. 1 serving of vegetable or fruits is 1 medium sized fresh fruit/vegetable; 1/2 cup of chopped, cooked, frozen, or canned fruit/vegetables;1/4 cup of dried fruit/vegetable. 1 cup = 250 ml

^b^ High income refers to the total annual household income more than $80,000; Middle income refer to the total annual household income lie between $30,000 and $79,999; low income refers to the total annual household income less than $30,000

^c^ Totals may not match as the “all” column includes responses from individuals who responded “not sure” to their income level

^d^r_s_ refers to Spearman correlation coefficient, which is used to measure the association degree between two ordinal variables (Correct = 1, Incorrect = 0; and Low income = 1, Middle Income = 2, High Income = 3)

The median score of risk factors was 9 correct out of 11. A weak-to-moderate and significant association was found between the participants’ awareness of risk factors and their income group *(r*_*s*_ = 0.237, *P* <  0.001). The lower the annual income, the lower the number of cancer risk factors were recognized. Specifically, weak but significant associations were noted for the following risk factors: drinking more than 1 unit of alcohol per day *(r*_*s*_ = 0.118, *P* <  0.001), being over 70 years old *(r*_*s*_ = 0.109, *P* = 0.001), and having a low-fiber diet (*r*_*s*_ = 0.089, *P* = 0.005, Table 2). Additionally, other characteristics were also associated with higher risk factor awareness scores (indicating they correctly identified more risk factors), including higher education (*r*_*s*_ = 0.231, P <  0.001), living in urban regions (*r*_*s*_ = 0.163, P <  0.001), and having a regular healthcare provider (*r*_*s*_ = 0.081, *P* = 0.010; analyses not shown).

### Beliefs about cancer treatment and outcomes, and screening

Composite beliefs scores were calculated for (1) beliefs about cancer treatment and outcomes, and for (2) beliefs about cancer screening. The items and scoring of these composite scores are displayed in Table 3. For beliefs about cancer treatment and outcomes, we dichotomized the composite variable into ‘more positive’ beliefs (median and above, or a score of 18 to 24) versus ‘more negative’ beliefs (below the median, or a score of 6 to 17). For beliefs about cancer screening, we dichotomized the composite beliefs score into ‘more positive’ beliefs (median and above, or a score of 32 to 40) versus ‘more negative’ beliefs (below the median, or a score of 8 to 31).

**Table 3:** Items Included in Beliefs Scores

Beliefs About Cancer Treatment & Outcomes	Beliefs About Cancer Screening
**Positively-framed Beliefs** ^a^	**Positively-framed Beliefs** ^c^
1. These days, many people with cancer can expect to live normal lives	1. Cancer screening could reduce my chances of dying from cancer.
2. Cancer can often be cured	2. Cancer screenings are now very routine tests
3. Going to the doctor as quickly as possible after noticing a symptom of cancer could increase chances of surviving	3. I would be more likely to participate in screening if my doctor told me how important it was
	4. Regular cancer screening would give me a feeling of control over my health
**Negatively-framed Beliefs** ^b^	**Negatively-framed Beliefs **^d^
4. Some people think that a diagnosis of cancer is a death sentence. To what extent do you agree or disagree with them?	5. I would be so worried about what might be found during screening, that I would prefer not to do it
5. Most cancer treatment is worse than the cancer itself	6. Cancer screening tests have a high risk of leading to unnecessary surgery
6. I would not want to know if I have cancer	7. If I have a healthy lifestyle, I don’t need to worry about having regular cancer screening
	8. Cancer screening is only necessary if I have symptoms

^a^ Strongly Disagree = 1, Disagree = 2, Agree = 3, Strongly Agree = 4

^b^ Strongly Disagree = 4, Disagree = 3, Agree = 2, Strongly Agree = 1

^c^ Strongly Disagree = 1, Disagree = 2, Neither Agree nor Disagree = 3, Agree = 4, Strongly Agree = 5

^d^ Strongly Disagree = 5, Disagree = 4, Neither Agree nor Disagree = 3, Agree = 2, Strongly Agree = 1

Logistic regression found no statistically significant associations between more positive vs more negative beliefs about cancer treatment and outcomes and age, ethnicity, having had a significant other with a history of cancer, or having had a first-degree relative with a history of cancer (see Table 4). The strongest association was that participants who had a history of cancer themselves had greater odds of having more positive beliefs than those without a history of cancer (OR = 3.40[2.15–5.37]). In terms of socioeconomic variables, compared to the low-income group, those in the high-income group had significantly higher odds of having more positive beliefs about cancer treatment and outcomes (OR = 1.67[1.01–2.75]). Similarly, compared to those without post-secondary education, those with post-secondary education had significantly higher odds of having more positive beliefs about cancer treatment and outcomes (OR = 1.52[1.04–2.22]). Compared with participants whose self-rated health was ‘poor or fair’, those with ‘very good or excellent’ health, but not those with ‘good’ health, had significantly higher odds of having more positive beliefs about cancer treatment and outcomes (OR = 2.58[1.63–4.08]).

**Table 4:** Multivariate logistic regression analysis for effect of sociodemographic characteristics on beliefs about cancer treatment and outcomes, and on beliefs about cancer screening

Variable	Beliefs about cancer treatment and outcomes (***n*** = 842^**b**^)		Beliefs about cancer screening (***n*** = 851^**b**^)	
	OR (95%CI)	P value	OR (95%CI)	P value
**Age group (years)**				
35–39	Reference		Reference	
40–44	0.96 (0.52–1.80)	0.909	0.78 (0.42–1.43)	0.419
45–49	0.85 (0.47–1.54)	0.602	0.90 (0.50–1.61)	0.726
50–54	1.21 (0.68–2.14)	0.520	0.81 (0.46–1.43)	0.476
55–59	1.02 (0.57–1.84)	0.937	1.06 (0.59–1.89)	0.846
60–64	1.09 (0.59–2.02)	0.775	0.95 (0.52–1.72)	0.859
65–69	1.06 (0.56–2.00)	0.865	0.96 (0.51–1.79)	0.899
70–74	1.29 (0.61–2.73)	0.502	0.76 (0.37–1.54)	0.444
**Gender**				
Female	Reference		Reference	
Male	1.28 (0.91–1.81)	0.158	1.08 (0.77–1.50)	0.656
**Ethnicity**				
Caucasian/white	Reference		Reference	
Other	0.87 (0.42–1.79)	0.697	**0.48 (0.24–0.94)**	**0.034**
**Annual household income group**				
Low income (<$30,000)	Reference		Reference	
Middle income ($30,000–$79,999)	1.45 (0.91–2.30)	0.116	0.82 (0.53–1.29)	0.401
High income ($80,000+)	**1.67 (1.01–2.75)**	**0.044**	1.10 (0.67–1.78)	0.714
**Highest education attained**				
No Post-Secondary	Reference		Reference	
Post-Secondary	**1.52 (1.04–2.22)**	**0.029**	0.98 (0.68–1.42)	0.930
**History of Cancer (Self)**				
No	Reference		Reference	
Yes	**3.40 (2.15–5.37)**	**< 0.001**	**2.03 (1.34–3.07)**	**< 0.001**
**History of Cancer (Significant other**^a^**)**				
No	Reference		Reference	
Yes	1.18 (0.76–1.85)	0.457	1.00(0.65–1.53)	0.996
Not sure	0.59 (0.11–3.23)	0.547	0.27 (0.05–1.60)	0.148
**History of Cancer (Close family**^a^**)**				
No	Reference		Reference	
Yes	0.99 (0.71–1.37)	0.946	1.18 (0.86–1.62)	0.309
Not sure	1.23 (0.26–5.91)	0.793	2.77 (0.53–14.38)	0.224
**Self-rated health**				
Poor or Fair	Reference		Reference	
Good	1.52 (0.97–2.40)	0.068	1.47 (0.95–2.28)	0.080
Very Good or Excellent	**2.58 (1.63–4.08)**	**< 0.001**	**2.13 (1.37–3.30)**	**< 0.001**
**Number of chronic diseases**				
0	Reference		Reference	
1–2	**1.40 (1.02–1.92)**	**0.035**	1.03 (0.76–1.40)	0.827
3 or more	1.14 (0.72–1.80)	0.572	0.99 (0.63–1.54)	0.960

Adjusted Model: Age + Gender + Ethnicity + Income Group + Education + Cancer History (Self) + Cancer History (Significant Other) + Cancer History (Close family) + Self-rated Health + Number of Chronic Diseases

^a^Close family refers to parents, siblings, children, etc.; Significant other refers to husband/wife, partner, etc.

^b^ Cases missing responses to any of the regression variables were excluded from the analysis

Logistic regression found no significant associations between more positive vs more negative beliefs about cancer screening and age, gender, income, education, number of chronic illnesses, or having had a significant other or close family member with a history of cancer (see Table 4). The only significant associations were for ethnicity, self-rated health, and history of cancer. Participants who have had cancer themselves had greater odds of having more positive beliefs about cancer screening than those never diagnosed with cancer (OR = 2.03[1.34–3.07]). Compared with participants whose self-rated health was ‘poor or fair’, those with ‘very good or excellent’ health, but not those with ‘good’ health, had significantly higher odds of having more positive beliefs about cancer screening (OR = 2.13[1.37–3.30]). Logistic regression also found that those who were non-Caucasian/white had significantly lower odds of having more positive beliefs about cancer screening (OR = 0.48[0.24–0.94]).

## Discussion

Measuring and understanding public awareness and beliefs about cancer could help in developing appropriate policies for cancer prevention and control. To our knowledge, this is the first study conducted to measure the public awareness and beliefs about cancer in Newfoundland and Labrador. Generally, the majority of participants had correct recognition of risk factors for cancer. However, we found poor awareness of the increased cancer risk associated with low-fiber diets, eating more red and processed meat diets, consuming alcohol, and being older. Income-level, education-level, urban geography (vs rural), and having a regular healthcare provider (vs not having one) all had statistically significant positive correlations with greater awareness of risk factors. Understanding perceived beliefs about cancer are important because fatalistic beliefs are linked to decreased uptake of screening and delayed presentation [25, 26]. Of the factors we studied, we found that income, education, self-rated health, number of chronic diseases, and a history of cancer all had associations which affected beliefs about cancer treatment and outcomes. Similarly, self-rated health, history of cancer, and ethnicity were the only factors affecting beliefs about cancer screening.

### Awareness of cancer risk factors

In our study, the majority of participants agreed with the fact that cigarette smoking (smoking and second-hand smoking) and family history can increase the risk of cancer, and a strong socioeconomic gradient was found in recognition of risk factors for cancer. People with a lower household income and a lower educational level were more likely to have a lower awareness of cancer factors (including older age, drinking alcohol, eating a low-fiber diet) than people with a high-level education and people with high household income; these findings were consistent with other previous studies [4, 27–30]. Furthermore, as is well known, the incidence rate for most cancers increases with age [1]. However, more than half the participants in our study thought people of any age were equally likely to be diagnosed with cancer. This surprising finding has been found elsewhere [4, 31].

While several risk factors were found to have greater awareness associated with higher income, the general level of awareness was still low for all income groups (particularly for these risk factors: older age, drinking alcohol, eating a low fiber diet, eating more red or processed meat). Therefore, while awareness campaigns may address a larger gap in knowledge and by targeting health education towards low- and middle- income groups, we believe this lack of awareness must be addressed across all socioeconomic groups.

It is important to note that public health campaigns focusing predominantly on behavioural health-promotion for disease prevention often fail to reach their goals, likely because they neglect the role of the social determinants of health [32]. Indeed, while behavioural and educational interventions are important, including the targets identified within this study—such as promoting cancer risk associated with low fiber diets, increased alcohol consumption, and eating red and processed meats—adequate economic resources are still required for individuals to put behavioural health strategies into action. As such, to improve disease prevention, it is imperative that governments improve the basic social and economic conditions of their citizens.

### Beliefs about cancer treatment, outcomes, and screening

Our study found a few sociodemographic characteristics which were associated with beliefs about cancer treatment and outcomes, but only a couple that were associated with beliefs about screening. We found that both income and education had statistically significant associations with beliefs about cancer treatment and outcomes. Those with post-secondary education had higher odds of having more positive beliefs about cancer treatment and outcomes than those without post-secondary education. Similarly, relative to the low-income group, those in the high-income group had greater odds of having more positive beliefs about cancer treatment and outcomes. These findings support the associations with income and education found elsewhere: population-based studies in Denmark and the UK found several associations between income or education and negatively-framed beliefs, though only a few small associations were found with the positively-framed beliefs [11, 12]. The UK study also found it was common to simultaneously hold both more positively-framed and more negatively-framed beliefs about cancer treatment and outcomes, particularly among those with lower levels of education [11].

Unexpectedly, we did not find any significant associations between beliefs about cancer screening and income or education. This may be considered a promising finding, suggesting that public health campaigns, education efforts, and access to knowledge is reaching adults in Newfoundland and Labrador in an equitable way and that socioeconomic inequities are not affecting beliefs about cancer screening.

Our study also showed that participants in better health had significantly more positive beliefs about cancer treatment and outcomes, as well as screening, than those who rated their health as ‘poor or fair’. This finding has been reported elsewhere [12]. This may be due to the fact that people in better health feel more confident about their health, whereas those who are in poorer health may feel worn down by their illness, hopeless, or as if they have less control. When compared to participants with no chronic diseases, we also found that those with 1–2 chronic diseases, but not those with 3 or more chronic diseases, had greater odds of having more positive beliefs about cancer treatment and outcomes. This interesting finding should be explored further.

In our regression analyses, we found that participants who have previously been diagnosed with cancer had higher odds of having more positive beliefs about cancer treatment and outcomes, and about cancer screening, than those never diagnosed with cancer. However, it is likely that this is influenced by a survivorship bias, where those who survived feel less fearful of cancer or fatalistic in their beliefs because they survived. Interestingly, Hvidberg et al. [12] found that, of the six beliefs about cancer treatment and outcomes they studied, only one had a statistically significant association with having a history of cancer, and it was so small as to almost be negligible. While this is in contrast with our findings, our studies measure this concept in slightly different ways, which may influence the results: whereas we looked at participants who ever vs never had a diagnosis of cancer, Hvidberg et al. [12] looked at those who had cancer within the last 10 years vs not within the last 10 years (or not at all).

We had expected that those who had a close family member or significant other diagnosed with cancer would be more primed by this experience to consider their own risk of cancer, and to thus engage in preventive and health-promoting behaviours. Interestingly, however, we failed to find an association between beliefs about cancer screening and having had a significant other or a close family member (first-degree relative) diagnosed with cancer. This suggests that those who are socially, emotionally, and physically close to those diagnosed with cancer are not significantly impacted in their beliefs about cancer treatment, outcomes, or screening. This is unexpected, as other studies have found associations between vicarious experiences of a cancer diagnosis in family and friends and illness representations, specifically in beliefs about causation [4, 33], as well as associations with beliefs about treatment and outcomes [12]. That said, Hvidberg et al. found the associations were primarily with negatively-framed beliefs, not positively-framed beliefs [12].

While beliefs about cancer treatment and outcomes were not associated with ethnicity, one important finding was that participants who identified as non-Caucasian/white ethnicity had half the odds of having more positive beliefs about cancer screening compared to those who identified as Caucasian/white. In other words, non-Caucasian/white participants had more negative beliefs about cancer screening than Caucasian/white participants. We recognize that grouping all non-Caucasian/white ethnicities into one ‘Other’ group fails to recognize the diversity of backgrounds, experiences, and beliefs represented in this category. Nonetheless, it is an important finding and future research is warranted to identify which groups specifically have less-positive beliefs about screening, why it is as such, and whether this impacts participation in screening programs.

### Strengths and limitations

The main strength of this study is that recruiting by Facebook-advertising allowed us to reach a large number of participants across the province at a low cost. Further, this is the first study of its kind conducted using a large sample to assess public cancer awareness and beliefs in Newfoundland and Labrador.

Nevertheless, some limitations of this study should be noted. First, as we used an online survey, we were not able to assess the response rate and make necessary comparisons between respondents and non-respondents. Second, since the majority of the current sample identified as Caucasian/white, conclusions drawn by ethnicity are constrained. However, the ethnic makeup of the sample (Caucasian/white vs Other) did mimic that of the general population of Newfoundland and Labrador. Third, all data in this study were collected and obtained through Facebook advertising, which was a non-random sampling method and may thus have introduced sampling bias. However, we employed purposive targeting to help improve the representativeness of the sample, which we determined was partially representative of the population [21]. Even then, given the self-selecting nature of participation, those who are more aware of cancer risks or are more concerned about their own risk of cancer, may have been more likely to respond to the survey compared to those who have low awareness or concern. Lastly, this study was a cross-sectional study and some participants’ information were self-reported, and thus the possibility of biases in the accuracy of recall information could not be eliminated.

## Conclusion

Given the low level of awareness for certain important risk factors among NL adults in our study, we believe that an important public health goal will be to improve awareness of these risk factors, as improved awareness can facilitate healthcare seeking and healthy behaviors, which may in turn reduce the burden of disease. Future research should seek to further elucidate how social determinants and other factors impact the ability of individuals to take knowledge about prevention and put it into action.

## Supplementary information

**Additional file 1.** Supplemental File 1. Survey Items.

## Data Availability

The datasets generated during and/or analysed during the current study are available from the corresponding author on reasonable request.

## Abbreviations

NL: Newfoundland and Labrador
ASIR: Age-standardized incidence rates
ASMR: Age-standardized mortality rates
ABC: Awareness and Beliefs about Cancer
CAM: Cancer Awareness Measure
