# Supplementary material for: Sociodemographics and their impacts on risk factor awareness and beliefs about cancer and screening: results from a cross-sectional study in Newfoundland and Labrador
Source: BMC Public Health. 2020 Oct 6;20:1513. doi: 10.1186/s12889-020-09616-2 (PMC7539438; doi:10.1186/s12889-020-09616-2)
Supplement: Supplementary file 1 — Additional file 1. Supplemental File 1. Survey Items. [file 12889_2020_9616_MOESM1_ESM.docx]

**Supplemental File 1. Survey Items**

This is supplemental file to the manuscript entitled “Sociodemographics and their impacts on risk factor awareness and beliefs about cancer and screening: results from a cross-sectional study in Newfoundland and Labrador” by Shi et al., published in BMC Public Health.

Several items (C1, C2, C3, C4, D1a, D1b, and D1c) in this instrument were adapted from the *Awareness and Beliefs about Cancer (ABC) Instrument* developed by Simon et al. (2011) in collaboration with the International Cancer Benchmarking Partnership and the Cancer Research UK Health Behaviour Research Center. We would also like to acknowledge the work of Robb et al. (2009) and the Cancer Research UK Health Behaviour Research Center in developing the *Cancer Awareness Measure (CAM),* as many of the questions in the *ABC Instrument* were developed initially for the *CAM Instrument.* The items in this supplemental file were reproduced with permission.

The footnotes in this file provide additional information on how certain items were scored and on any major changes which were made from the original instruments.

**Prevention of Cancer with Lifestyle Modifications: bridging research, health awareness, and practice in Newfoundland and Labrador**

**CONSENT AND ELIGIBILITY**

**Do you consent to participating in this study?**

By selecting “Yes” and click "Next," you are consenting to be in this study. It tells us you understand the information about the research study. When you select “yes” and consent to be a participant in this study, you are not giving up your legal rights. Researchers or agencies involved in this research study still have their legal and professional liabilities

 Yes

 No

**Eligibility Question 1.** Are you between 35 and 74 years old?

 Yes

 No

**Eligibility Question 2.** Are you a resident of Newfoundland & Labrador and have lived here for at least the past 2 years?

 Yes

 No

**SECTION A. *General Physical and Social Well-Being***

*We would like to start by asking you a few questions about your health.*

A1. **Would you say that your general health is:** *(select one)*

5= Excellent

4= Very Good

3= Good

2= Fair

1= Poor

A2. **Thinking about the amount of stress in your life, would you say that most of your days are:** *(select one)*

5= Not at all stressful

4= Not very stressful

3= A bit stressful

2= Quite a bit stressful

1= Extremely stressful

A3. *^[[1]](#footnote-0)^* **Please list any “long-term” chronic health conditions that you have been diagnosed with by a health professional**: *(Long-term means they have lasted or are expected to last at least 6 months or more).*

_______________________________________

A4. **Have you ever been diagnosed with cancer?**

2= Yes

1= No

A5. **We would like to know if any of your friends or family members have ever been diagnosed with cancer:**

|  |  | **Yes** | **No** | **Not sure** |
| --- | --- | --- | --- | --- |
| 5a. | A significant other (e.g. husband/wife, partner, etc.) | =2 | =1 | =9 |
| 5b. | A close family member (e.g. parents, siblings, children, etc.) | =2 | =1 | =9 |
| 5c. | Other family member (e.g. aunt/uncle, grandparents, cousins, etc.) | =2 | =1 | =9 |
| 5d. | A close friend | =2 | =1 | =9 |
| 5e. | An acquaintance | =2 | =1 | =9 |

**SECTION B. *Health Care***

B1. **Do you have a regular health care provider? By this, we mean one health professional that you regularly see or talk to when you need care or advice for your health?**

2= Yes

1= No

**SECTION C. *Cancer Awareness and Beliefs***

C1. *^[[2]](#footnote-1)^* **People hold different beliefs about cancer. What is your opinion about the following common beliefs:** *(check the box that best reflects your agreement with each statement)*

| **Beliefs About Cancer (Treatment and Outcomes)** | | | **Strongly Disagree** | **Disagree** | **Agree** | **Strongly Agree** |
| --- | --- | --- | --- | --- | --- | --- |
| 1a. | | These days, many people with cancer can expect to live normal lives | 1= | 2= | 3= | 4= |
| 1b. | | Most cancer treatment is worse than the cancer itself | 4= | 3= | 2= | 1= |
| 1c. | | I would not want to know if I have cancer | 4= | 3= | 2= | 1= |
| 1d. | | Cancer can often be cured | 1= | 2= | 3= | 4= |
| 1e. | | Going to the doctor as quickly as possible after noticing a symptom of cancer could increase chances of surviving | 1= | 2= | 3= | 4= |
| 1f. | Some people think that a diagnosis of cancer is a death sentence. To what extent do you agree or disagree with them? | | 4= | 3= | 2= | 1= |

C2.**^[[3]](#footnote-2)^ The following is a list of things that may or may not change a person’s risk of getting cancer. For each of the following items, please indicate whether you think that it would increase risk, decrease risk, or have no effect on a person’s risk of getting cancer:**

| **Cancer Risk Factor Awareness** | | **Decreases Risk** | **No Effect** | **Increases Risk** |
| --- | --- | --- | --- | --- |
| 2a. | Smoking any cigarettes at all | 0= | 0= | 1= |
| 2b. | Exposure to another person's cigarette smoke | 0= | 0= | 1= |
| 2c. | Drinking more than 1 unit of alcohol per day | 0= | 0= | 1= |
| 2d. | Eating more than 5 servings of fruits and vegetables per day | 1= | 0= | 0= |
| 2e. | Eating red or processed meat once per day or more | 0= | 0= | 1= |
| 2f. | Being overweight | 0= | 0= | 1= |
| 2g. | Getting sunburnt more than once as a child | 0= | 0= | 1= |
| 2h. | Being over 70 years old | 0= | 0= | 1= |
| 2i. | Having a close relative who has had cancer | 0= | 0= | 1= |
| 2j. | Infection with HPV (Human Papillomavirus) | 0= | 0= | 1= |
| 2k. | Having a diet low in fibre | 0= | 0= | 1= |

**C3. Over the next year, which of these groups of people do you think is most likely to be diagnosed with cancer?** *(select one)*

1= 30 year olds

2= 50 year olds

3= 70 year olds

4= People of any age are equally likely to be diagnosed with cancer

**C4. If you notice symptoms that you think might be serious, how soon would you make an appointment to see a doctor or health care professional to discuss it with them?** *(select one)*

1= Within a day

2= Within a week

3= Within a month

4= Longer than a month

5= I would not make an appointment

**SECTION D. *Cancer Screening Awareness and Beliefs***

D1.^[[4]](#footnote-3)^ **People hold different beliefs about cancer screening, and their behaviours influenced by different factors. How much do you agree or disagree with each of the statements below?** *(check the box that best reflects your agreement with each statement)*

|  | | **Strongly Disagree** | **Disagree** | **Neutral** | **Agree** | **Strongly Agree** |
| --- | --- | --- | --- | --- | --- | --- |
| 1a. | I would be so worried about what might be found during screening, that I would prefer not to do it. | 5= | 4= | 3= | 2= | 1= |
| 1b. | Cancer screening is only necessary if I have symptoms. | 5= | 4= | 3= | 2= | 1= |
| 1c. | Cancer screening could reduce my chances of dying from cancer. | 1= | 2= | 3= | 4= | 5= |
| 1d. | If I have a healthy lifestyle, I don’t need to worry about having regular cancer screening | 5= | 4= | 3= | 2= | 1= |
| 1e. | Cancer screenings are now very routine tests | 1= | 2= | 3= | 4= | 5= |
| 1f. | Cancer screening tests have a high risk of leading to unnecessary surgery | 5= | 4= | 3= | 2= | 1= |
| 1g. | Regular cancer screening would give me a feeling of control over my health | 1= | 2= | 3= | 4= | 5= |
| 1h. | I would be more likely to participate in screening if my doctor told me how important it was | 1= | 2= | 3= | 4= | 5= |

**SECTION H*. Demographic Information***

**H1**. **What gender do you identify as?**

1= Female 2= Male 9= Other user-missing= Prefer not to say

**H2.** **In what age category do you belong?** *(select one)*

1= 35 – 39 2= 40 – 44 3= 45 – 49 4= 50 – 54

5= 55 – 59 6= 60 – 64 7= 65 – 69 8= 70 – 74

**H3.** **Which best describes your ethnic group?** 1= Caucasian/white 2= Other

**H4**. **What is your body weight?** (please specify kilograms or pounds)

*For example, if you weigh 165 pounds, please write either "165 pounds" OR "74.8 kilograms^[[5]](#footnote-4)^*

_____________________

**H5.** **What is your body height?** (please specify centimetres or feet and inches)

*For example, if you are 5 feet 11 inches, write "5 feet 11 inches" OR "180 centimetres"*

_____________________

**H6. What are the first three characters of your residential (home) postal code?^[[6]](#footnote-5)^**

__________

**H8**. **Are you currently living with a partner or significant other (such as a husband/wife, common-law partner, or romantic other)?** *(select one)*

1= No, I am not living with a partner or significant other

2= Yes, I am living with a partner or significant other

**H9.** **What is the highest level of education you have completed?** *(select one)*

1= Did not complete high school

2= High school

3= College diploma or university degree

4= Graduate, Postgraduate, or Professional Degree

**H10. Which of the following categories best describes your total annual household income before taxes?** *(select one)^[[7]](#footnote-6)^*

1= Less than $12,000 3= $60,000 to $79,999

1= $12,000 to $29,999 3= $80,000 to $99,999

2= $30,000 to $49,999 3= More than $100,000

2= $50,000 to $59,999 9= Don’t know

***This is the end of the questionnaire. Thank you very much for participating.***

1. We counted the number of conditions listed and entered this as a number. We then converted this into a categorical variable: none (=0), one to two (=1), 3 or more (=2) [↑](#footnote-ref-0)
2. For the Total Beliefs About Cancer score, we did a raw total of each item C1a-f. The original ABC instrument recommends combining items 1a, 1d, and 1f using a weighted score = (1a)*(0.3756) + (1d)*(0.7024) + (1f)*(0.2501). We decided against this, choosing to calculate a arithmetic sum of scores on each of the 6 items for the total score. [↑](#footnote-ref-1)
3. The original ABC (Awareness and Beliefs about Cancer) instrument used a different scoring scale (“Strongly Agree”; “Tend to Agree”; “Tend to Disagree”; and “Strongly Disagree”) and we modified this to more simplistic response options (“Increases Risk”; “No Effect”; and “Decreases Risk”). Correct Responses were scored as “1” and incorrect responses were scored as “0”.
   One item was added to our instrument that was not included in the original ABC instrument: “having a diet low in fibre.”
   When transcribing our instrument from our draft in Microsoft Word to the online version, one item was inadvertently omitted from the final online instrument: “Engaging in 30 minutes of moderate physical activity 5 times per week or more.” [↑](#footnote-ref-2)
4. The original ABC (Awareness and Beliefs about Cancer) instrument used a different scoring scale (“Strongly Agree”; “Tend to Agree”; “Tend to Disagree”; and “Strongly Disagree”) [↑](#footnote-ref-3)
5. Height and weight were used to calculate BMI [↑](#footnote-ref-4)
6. Postal codes with a ‘0’ as the second character, based on Canada Post’s definition of rural, were assigned as “Rural”(=1) and all other postal codes were considered “Urban”(=2) [↑](#footnote-ref-5)
7. Response categories for income were combined into three categories: <$30,000 “Low Income” (=1), $30,000-59,999 “Middle Income” (=2), and $60,000+ “High Income” (=3) [↑](#footnote-ref-6)
